# Predicting the Topological and Transport Properties in Porous Transport Layers for Water Electrolyzers

**DOI:** 10.1021/acsami.3c12345

**Published:** 2023-11-10

**Authors:** Jiang Liu, Felix Kerner, Nicolas Schlüter, Daniel Schröder

**Affiliations:** † Institute of Energy and Process Systems Engineering, Technische Universität Braunschweig, Langer Kamp 19B, 38106 Braunschweig, Germany; ‡ Battery LabFactory Braunschweig (BLB), Technische Universität Braunschweig, Langer Kamp 19, 38106 Braunschweig, Germany

**Keywords:** porous transport layer, water electrolyzer, topology, electrical conductivity, thermal conductivity

## Abstract

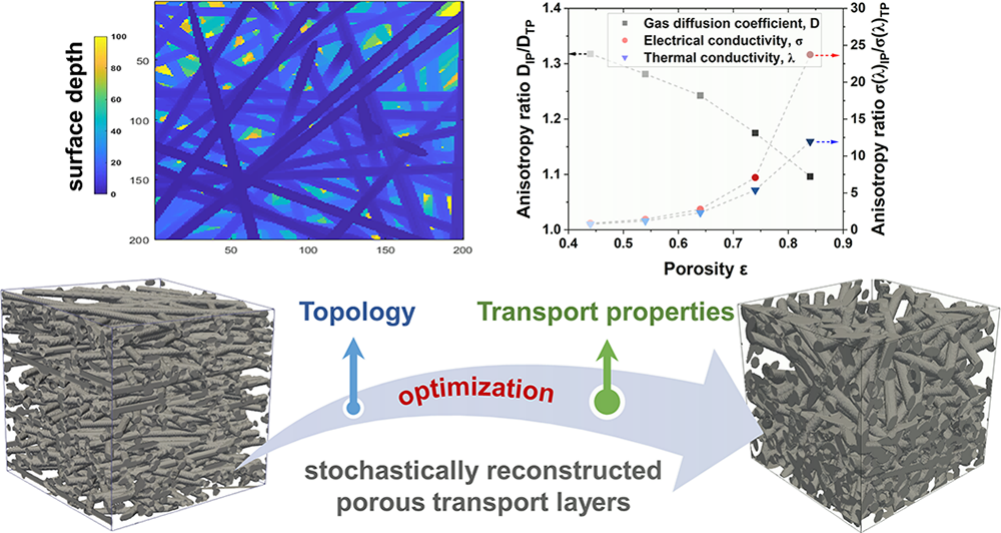

The porous transport layer (PTL) in polymer electrolyte membrane (PEM) electrolyzers governs the overall efficiency. Its structural, thermal, and electronic properties determine how effortlessly the gases can be produced and can exit the PEM electrolyzer. In this study, we apply a stochastic reconstruction method for titanium felt-based PTLs to generate PTLs with different porosity, fiber radii, and anisotropy parameters. The morphology and topology of these PTLs are numerically characterized, and transport properties, such as gas diffusion coefficients and electrical and thermal conductivity, are computed via pore-scale modeling. Customized graded PTLs are proposed, exhibiting the optimal topology and bulk structure for the removal of gases, the conductance of electrons, and the transport of heat. The results indicate that the surface and transport properties of PTLs can be tailored by certain morphology parameters: PTLs with lower porosity and smaller fiber radii feature a more sufficient interfacial contact and superior electrical and thermal conductivity. Lowering the anisotropy parameters of PTLs results in a slight loss of interfacial contact but a substantial increase in the electrical and thermal conductivity in the through-plane direction. We outline that the design of PTLs should be differentiated depending on the operating conditions of electrolyzers. For nonstarvation conditions, PTLs should feature low porosity and small fiber radii, whereas for starvation conditions, PTLs should feature high porosity, low anisotropy parameters, and small fiber radii. Furthermore, graded PTLs with enhanced structural and transport properties can be developed by customizing the porosity, fiber radius, and fiber orientation.

## 1 Introduction

The combined utilization of polymer electrolyte membrane (PEM) electrolyzers and PEM fuel cells is promising for future energy supply on a large scale. Especially when PEM electrolyzers are coupled with renewable energy sources, they can effectively resolve both issues with carbon emissions and intermittency. However, the commercialization of PEM electrolyzers is still at an early stage, and further research and development is needed.^1^ One of the more attractive technical measures to speed up commercialization is to increase the operating current density of the electrolyzer. To achieve this goal, optimization of mass transport of the product gases and decrease of ohmic losses originating from the porous transport layers (PTLs) is required.^2,3^ Similar enhancements have been implemented for applications such as fuel cells and flow batteries.^4−7^

During the electrochemical reaction within PEM electrolyzers, the transport of reactant water, product gas, protons, as well as the conduction of electricity and heat, occur almost simultaneously. The PTL, especially the anodic PTL, is essential for facilitating the aforementioned interlinked phenomena. The PTL provides pathways for the reactant water to the catalyst layer and for the product oxygen to the flow channel. While facilitating mass transport, it also needs to maintain good electrical and thermal conductivity and provide sufficient mechanical support.^8^ These demanding requirements and the high electrochemical potential make the use of mechanically stable and corrosion-resistant materials like titanium (Ti) obligatory for the anodic PTL.^9^ Despite the excellent electrochemical stability of Ti leading to its widespread use, the electrochemical losses associated with the structure of PTLs made of titanium materials are still not negligible. The pore network in PTLs usually facilitates the transport of the phases liquid (water) and gas (oxygen or hydrogen). On the contrary, the solid network needs to facilitate electrical and thermal conduction at the same time, which makes the design of PTL structures a topic of opposite poles. In general, high porosity and pore size could facilitate the transport of oxygen and water within the PTL, thereby reducing mass transport losses. However, high porosity and pore size could also result in inadequate solid network connections and high surface roughness, which in turn leads to reduced catalyst utilization, high ohmic resistances, and increased interfacial resistances.^10^ This issue is further complicated by the way PTL structures are manufactured, e.g., sintered powder structures,^11,12^ felt structures,^10,13,14^ and mesh structures.^15^

Therefore, general PTL design guidelines that weigh mass transport losses against ohmic losses need to be established before optimal designs can be proposed for industry. Extensive research on the PTL structure and electrolyzer performance has emerged in recent years.^8,9,11,15−26^ These research studies can be grouped into two categories. The first category contains work on the impact of PTL structures on the two-phase flow,^27−32^ while the second category focuses more on the relationship between the PTL structure and electrolyzer efficiency.^22,23,25,33−35^

Nouri-Khorasani et al.^27^ numerically investigated the formation and growth of oxygen bubbles inside the PTLs and evaluated their impact on the overpotential. They also modeled the effect of a single parameter, the PTL pore size, on the growth and detachment of oxygen bubbles. Arbabi et al.^28^ and Lee et al.^29^ applied a microfluidic platform to observe the transport characteristics of oxygen within a two-dimensional PTL. They used this platform to study and compare oxygen invasion patterns within three different types of PTL structures and to determine the effect of the feedwater rate on oxygen propagation. Hinebaugh et al.^30^ and Lee et al.^2,31,36^ applied a numerical method known as pore network modeling (PNM) to investigate the mass transport within PTLs. In their work, several PTLs with different structural characteristics were reconstructed by experimental and stochastic methods for the analysis of bulk structural and interfacial differences. Subsequently, the effect of porosity, pore and throat size, and gradient porosity on the gas saturation, and permeability was explored by PNM.

In addition to the PNM, the Lattice Boltzmann Method (LBM) is an often-used tool for observing the two-phase flow within the PTL and other porous materials due to its superior two-phase flow solver.^37,38^ Peng et al.^39^ analyzed the effect of PTL structures on each overpotential and concentration distributions for oxygen and water by using LBM in combination with electrochemical experiments and compared the bulk structural and interfacial differences of PTLs with varied porosity and pore sizes. Bhaskaran et al.^40^ used X-ray tomographic microscopy (XTM) to experimentally characterize the structures of several graded PTLs and simulated the transport of oxygen by LBM. In their simulations, the effect of the pore size distribution (PSD) of different PTLs on the two-phase distribution was evaluated, and the effect of the computational domain size and boundary conditions on the simulated oxygen transport was analyzed. Similarly, the LBM has been used as a powerful tool to obtain gas concentration distributions and invasion patterns within the PTL in the work of Paliwal et al.^41^ and Satjaritanun et al.^42^ Besides numerical observations, experiments such as structure characterization,^33^ optical imaging,^43^ X-ray radiography,^44^ and neutron imaging,^45^ were also conducted to characterize the porous structure and investigate the flow regime and mass transport.

Studies exploring the relationship between the PTL structure and PEM electrolyzer performance provide more direct and effective insight into the application of PTLs.^11,25,26,39,46−52^ For instance, in the work of Majasan et al.,^46,53^ several PTL structures were characterized using scanning electron microscopy (SEM), mercury intrusion porosimetry (MIP), and X-ray computed microtomography (XCT) to obtain the structural information such as the porosity and mean pore diameter (MPD). Subsequently, polarization curves and impedance spectra were measured and used to analyze the performance of electrolysis cells equipped with these PTLs. The results indicate a strong correlation of performance with the MPD of PTLs and suggest that a maximum interfacial area between the PTL and the catalyst layer (CL) is always desired. Kang et al.^54^ compared the performance of PEM electrolyzers, each applying different PTL types (Ti felt, sintered Ti powders, and carbon paper) by means of a mathematical model. A detailed electrochemical loss breakdown was used to analyze the activation, ohmic, and mass transport losses of each PTL type over a range of conditions. Some work was also devoted to exploring the comprehensive impact of PTL thickness on the structural morphology, transport properties, two-phase flow, and electrochemical performances.^33,50,55−57^ It was generally agreed that for specific operating conditions, there would usually be an optimum thickness to weigh the individual losses. In the experiments of Weber et al.,^55^ they deduced that the optimal PTL thickness corresponds to around half of the flow field land size.

Numerous studies have been carried out to elucidate the two-phase flow regimes within PTLs and to reveal the relationship between the PTL structure and the performance of PEM electrolyzers. These studies provide an irreplaceable contribution to improving the efficiency of PEM electrolyzers and accelerating the commercialization process. It must be acknowledged, however, that further, more detailed, and comprehensive research is still essential, in particular, focusing on the link between the structural and transport properties of PTLs.^13,26,58^ Current research on this aspect and data on PTL transport properties are very scarce and not sufficiently comprehensive. The work by Majasan et al.^53^ and by Maier et al.^56^ correlated the performance of PEM electrolyzers with PTL structures. However, it was not investigated how different structures change the transport properties of PTLs and thus affect the electrolyzer performance. This intermediate link (structural-transport properties) should not be underestimated, as it provides the most direct and valuable guidelines to optimize PTL structures in order to reduce losses and improve the performance. The paradoxical nature of oxygen transport through pores and electrical/thermal conduction through solid materials puts the manufacturers of PTLs in a dilemma. The interfacial resistances brought by the inadequate interfacial contact between PTLs and CL should not be overlooked. These have led to the fact that the knowledge and optimization of PTL are still in the exploratory stage. Additionally, most of the existing studies have focused on sintered Ti powder-based PTLs, which has led to an even greater lack of research and data on Ti felt-based PTLs. Only Zielke^10^ and Schuler et al.^13^ provided limited data on Ti felt-based PTLs.

Therefore, a comprehensive parametric study of the relationship between PTL structural and transport properties is imperative. Elucidating the relationship and developing high-performance PTLs is of great significance for industry applications. High-performing PTLs are essential for the next generation of high-performance, low-cost PEM electrolyzers. Advancing PTLs is conducive to driving further improvements in electrolysis efficiency as well as the affordability of hydrogen costs, facilitating the widespread use of hydrogen as a clean energy carrier. At the same time, fully understanding property-structure-relation for PTLs might lead to improvements at the industrial scale. A thorough knowledge of the PTL structure will also help to generalize it to other fields where similar porous media are studied, such as energy engineering, chemical engineering, civil engineering, biomedicine, etc. In view of the Sustainable Development Goals (SDGs), improving the application of electrolyzers and fuel cells will have a positive impact on clean energy, sustainability, and the global transition toward more environmentally friendly power generation and energy storage technologies.

In our earlier work, we investigated the oxygen transport performance within PTLs for fixed and only selected sets of structural parameters.^59^ To further understand the functionality and variability of these PTLs, the herein presented work focuses on characterizing the topological and morphological features of PTLs. We elucidate a fundamental correlation between the gas diffusion coefficients and electrical and thermal conductivity in PTLs. These features are of strategic importance for reducing ohmic resistances and interfacial resistances. Specifically, a stochastic reconstruction method is employed to generate a series of felt PTLs with different porosity, fiber radii, and anisotropy parameters. The morphology (porosity and mean pore size etc.) and topology (surface roughness and specific surface area etc.) of these PTLs are subsequently characterized to obtain the structural information, and the transport properties are calculated by pore-scale modeling (PSM) to obtain the relationship between structural and transport properties. The results are validated by experimental measurements from the literature to demonstrate the feasibility and reliability of our approach. Finally, the above results are utilized for a more applied analysis, aiming to develop graded PTLs with enhanced structural and transport properties than the single-layer PTL structure. These graded PTLs are expected to exhibit the optimized performance in actual electrolyzers. This work not only provides a reliable research framework to explore the transport properties of PTLs but also provides in-depth insights into further optimizing the design and manufacture of PTLs and improving the performance of PEM electrolyzers.

## 2 Methodology

In this work, all steps were accomplished by numerical methods. First, a stochastic reconstruction method was used to generate Ti felt-based PTLs with various structural features, and subsequently, the morphological and topological characteristics of these PTLs were obtained by numerical characterization. Finally, the transport properties including the gas diffusion coefficients and electrical and thermal conductivity of these materials were calculated by PSM.

### 2.1 Stochastic Reconstruction

The stochastic reconstruction method was implemented in MATLAB R2021b and employed to generate the 3D PTLs. Usually, Ti felt-based PTLs are stacked fibers. Hence, the underlying logic of this method is to generate fibers one by one at random positions in the preset computational domain until the target porosity is met. Typically, both the structural and transport properties of commercial felt PTLs exhibited a strong anisotropy in the in-plane (IP) and through-plane (TP) directions. This correlation is because the fibers were generated layer by layer, which means that all fibers were stacked in the *x*–*z* (the in-plane) coordinate system, as shown in Figure S1 (Supporting Information; fibers shown in blue). Hence, the anisotropy parameter β (a positive value used to customize the orientation of fibers) was introduced in the stochastic reconstruction algorithm. In the herein applied method, by changing the value of β, fibers were also allowed to grow along the TP direction according to the probability density function in eq 1 and thus formed an angle with the IP direction, which was termed as the polar angle θ.
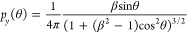
1

As shown in Figure S1, when β → 1, the most fibers will be evenly distributed throughout the whole domain, with most fibers at a 45° angle to the IP; when β → + ∞, most fibers will be stacked in the IP; and when β → 0, more fibers will be stacked in the TP, perpendicular to the CL.

**Figure 1:**
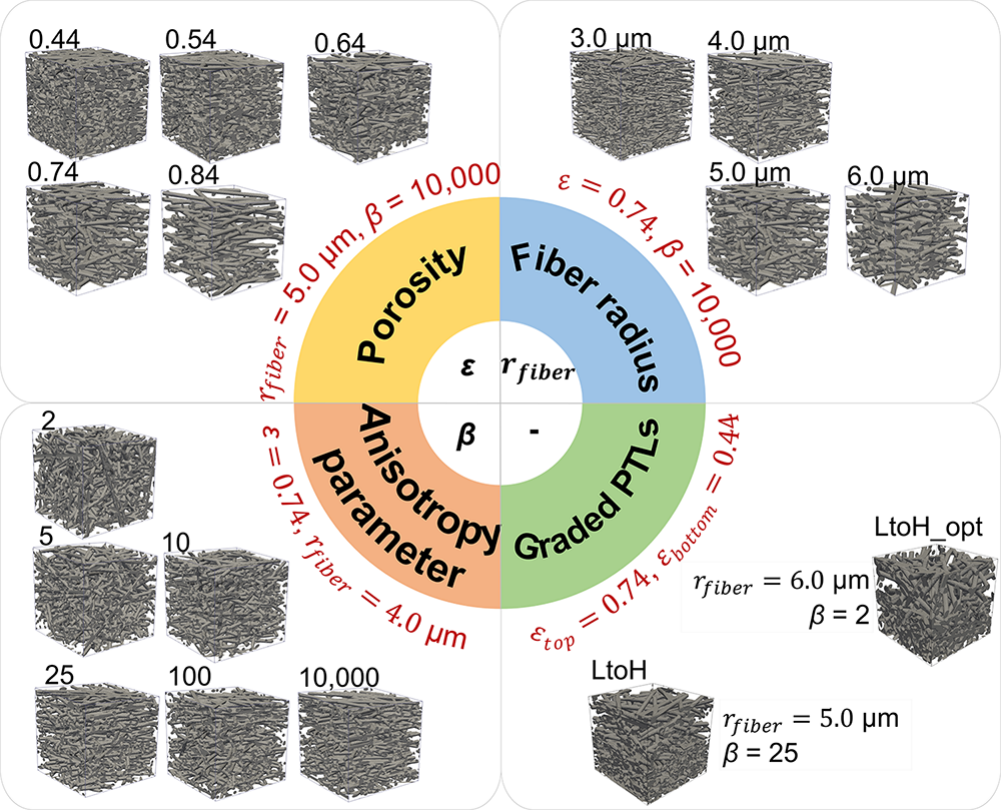
3D renderings of the reconstructed PTLs used within this work with different porosity (ε), fiber radii (*r*_fiber_), anisotropy parameters (β) indicated, and the graded PTLs (LtoH and LtoH_opt) with different fiber characteristics. Both graded PTLs include top and bottom layers with different porosity. LtoH denotes the configuration of the porosity gradient from low to high in the direction from the catalyst layer to the bipolar plate.

Usually, the representative elementary volume (REV) defining the smallest element which adequately represents the properties of the PTLs should be determined in numerical observations to minimize the computational cost. Based on previous experience^13,60^ and our test,^59^ a REV of 200 × 200 × 200 μm^3^ is efficient for the PTL representation. In this work, various PTLs with different porosity, fiber radii, and anisotropy parameters were reconstructed, as shown in Figure 1. In order to clearly distinguish between the PTLs, they were named after their main differences. For example, P44 with a porosity of 0.44 is one of the P-series PTLs featuring the same fiber radius and anisotropy parameter but a different porosity; R3 is the PTL with a fiber radius of 3 μm; B2 is the PTL with an anisotropy parameter of 2.

To validate the stochastic reconstruction method, a PTL with a porosity ε of 0.55 and fiber diameter *d*_fiber_ of 11.0 μm based on the experimental measurements by Schuler et al.^13^ was reconstructed. As shown in Table 1, the mean pore diameter *d*_*m*–pore_ of the reconstructed PTL agrees well with the experimental measurement. For more information on this reconstructed experimental PTL, we refer readers to previous work.^59^ In addition to the morphological/bulk properties, the topological and transport properties were also validated to prove the feasibility and validity of the numerical method in this work, which will be discussed in the following subsections.

**Table 1:** Comparison of Morphological, Topological, and Transport Properties between the Experimental and Reconstructed PTLs^a^

PTL	ε	*d*_fiber_	*d*_m–pore_	*R*_m_	*R*_SA_ (10 μm)	τ_IP/TP_	σ_IP/TP_	λ_IP/TP_
unit	%	μm	μm	μm	m^2^/m^2^_geo_	-	10^5^ S/m	W/mK
Schuler et al.^13^	55.0	11.0	20.6	21.7	1.1	1.7/1.6	6.4/5.4	6.0/5.9
reconstructed PTL	55.0	11.0	21.8	7.2	1.1	1.7/2.2	6.6/5.4	6.1/5.9

a The porosity ε, pore *d*_m–pore_ and fiber diameters *d*_fiber_, mean surface roughness *R*_m_, and specific surface area *R*_SA_ at a depth of 10 μm are listed. The tortuosity τ, effective electrical conductivity σ, and thermal conductivity λ in both IP/TP directions are shown.

### 2.2 Surface Roughness Characterization

The morphological properties of a PTL typically include the porosity, the size of pores and fibers/grains, and their size distributions, which can primarily affect mass transport losses; while the topology that captures the surface features contributes significantly to the losses for the electrochemical reactions during electrolysis.^61,62^ The surface roughness and the specific surface area are two commonly used terms to evaluate the characteristics of the PTL/CL interface.^2,13^

**Figure 2:**
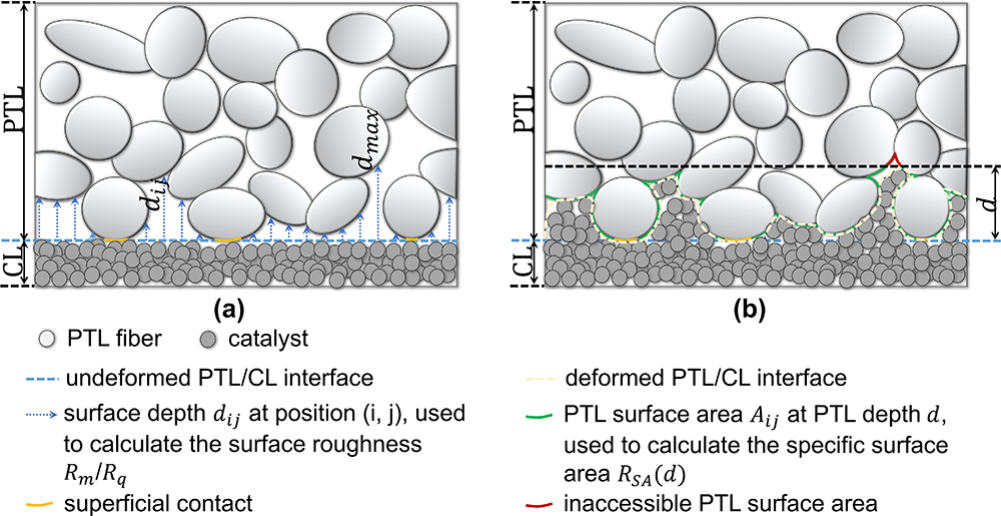
2D schematic of the PTL topological properties. (a) Undeformed interface. (b) Deformed interface. Adapted from the study of Schuler et al.^13^

As shown in Figure 2a, the surface depth *d*_*ij*_ at position (*i*,*j*) is defined as the distance from the PTL/CL interface to the solid material (along the direction normal to the interface). For the PTL samples in this work, all of the *d*_*ij*_ values formed a M × N (200 × 200) matrix, where the maximum depth value was termed as *d*_max_. According to eq 2, the mean surface roughness *R*_m_ was calculated based on this surface depth matrix. A high value of *R*_m_ usually indicates a coarse surface. Similarly, the root-mean-square roughness *R*_q_ in eq 3 was also introduced here to provide another parameter and more insight into the roughness.
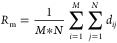
2
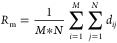

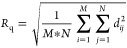
3

### 2.3 Specific Surface Area Characterization

The CL in an assembled electrolysis cell/stack inherently is pressed into the open pores of the PTL at its outer surface. Thus, the CL will not only be in superficial contact at the PTL/CL interface but also in the open pores of the PTL, as shown in Figure 2b. Therefore, the potential maximum contact area (the green curves in Figure 2b) is used in this work to characterize the interfacial contact between the PTL and the CL. The specific surface area *R*_SA_(*d*) was introduced and calculated by the following equation:
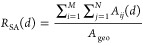
4where *A*_geo_ and *A*_*ij*_(*d*) are the active area and the surface area of the PTL respectively. *R*_SA_(*d*) represents the ratio of the potential maximum contact area at a certain PTL depth (*d*_PTL_) to the active PTL surface area. The specific surface area *R*_SA_ is a function of the PTL depth *d*_PTL_. For a given depth, a higher *R*_SA_ value usually indicates better interfacial contact.

The topological properties of the PTL reconstructed from experimental work were also calculated for comparison with experimental measurements. The results in Table 1 show that the values for *R*_SA_ are in good agreement, but are not a total match. Presumably, the reason for this deviation may be that the assumption that fibers were allowed to cross and overlap during the reconstruction led to an underestimation of the surface roughness of the PTL. However, the trends in roughness as a function of the PTL porosity in subsequent studies were consistent with experimental observations. This trend also demonstrates the feasibility of the method applied in our work.

### 2.4 Transport Property Calculation via PSM

In this work, pore-scale modeling was implemented to calculate the effective transport properties of dry PTLs, including the electrical conductivity σ, thermal conductivity λ, and also the gas (oxygen) diffusion coefficient *D* for reference. The PSM based on the finite volume method (FVM) resolves the transport processes by solving the governing equations. In this method, a steady-state condition and binary diffusivity is assumed. Knudsen diffusion is neglected due to large pores in PTLs. Single-phase conditions for dry PTLs are considered and therefore the effect of different water/gas contents is not taken into account. The transport of gas is solely driven by diffusion, while surface diffusion and gas adsorption are not considered. The PSM method was widely used for solving transport problems in porous structures. Only the essential equations are presented here, and the reader is referred to previous studies for more details on the formulas.^63−68^ The Fick’s first law describing the conservation of gas can be written as
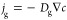
5where *j*_g_ is the diffusion flux of the gas, *c* is the gas concentration, and *D*_g_ is the gas diffusion coefficient.

To calculate the electrical and thermal conductivity, Ohm’s law in eq 6 and Fourier’s law in eq 7 are employed to resolve the electron and heat flux, respectively.
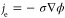
6
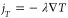
7

The *j*_e_ and *j*_*T*_ are the electron and heat flux, σ and λ are the electrical and thermal conductivity, and ϕ and *T* are the electric potential and temperature. By calculating the fluxes *j* of all these species, the effective transport properties *M*_eff_ can be computed according to the following equation:
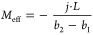
8where *b*_1_ and *b*_2_ are the prescribed boundary conditions, *L* is the domain length. In order to calculate the transport properties in different PTL directions, varied boundary conditions were used in PSM. Dirichlet boundary and periodic boundary conditions were selected for the direction of interest and the lateral directions, respectively. For a given temperature of 50 °C,^13^ the electrical and thermal conductivity of the PTL material Ti were assumed as 2.38 × 10^6^ S/m and 21.9 W/mK, and the thermal conductivity of H_2_O was assumed to be 0.6 W/mK.

The tortuosity τ is an intrinsic property of porous materials, which is governed by their microstructure. It can also be calculated as^14^
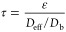
9where ε is the porosity of the PTL, *D*_eff_ is the effective oxygen diffusion coefficient, *D*_b_ is the oxygen bulk diffusion coefficient, and the value of *D*_eff_/*D*_b_ is termed as normalized effective oxygen diffusion coefficient *D*.

We applied the PSM then to compute the transport properties of the previously reconstructed PTL. As shown in Table 1, the electrical σ and thermal conductivity λ in all directions and the tortuosity τ in the IP direction are in very good agreement with the experimental results. The tortuosity in the TP direction deviates slightly from the experimental measurement, which is due to the more complex and tortuous structure of the reconstructed PTL in the TP direction caused by the crossing and overlapping of fibers.

## 3 Results and Discussion

The goal of this work is to correlate the morphology with the topology and transport properties of PTLs, providing insights into optimizing the design of PTLs for high-performance electrolyzers. In this section, the morphological and topological properties of PTLs with different structural features were first characterized. Subsequently, the transport properties of these PTLs were calculated by PSM, and the potential relationship between structural and transport properties was determined. Eventually, two customized graded PTLs were developed, which were designed in the computational procedure to exhibit optimized performance.

### 3.1 Morphology

The morphological properties of all the reconstructed PTLs are shown in Table 2. The second to the fourth columns in this table show the basic structural parameters of these PTLs, and the others were calculated by numerical characterization. It shows that with the increase of porosity, fiber radius, and anisotropy parameter, the mean pore size *r*_m–pore_ increases. And within the observed values, the effect of the porosity on the variation of mean pore size is more severe than for the fiber radius and anisotropy parameters, which is consistent with the conclusions for the sintered powder PTLs.^2^ The detailed pore size distribution of these PTLs is shown in Figure S2. This finding implies that in practical applications of PTLs, the porosity should be chosen as the critical design parameter to adjust the PTL structure, while the fiber radius and anisotropy parameter shall be used for fine-tuning.

**Table 2:** Morphological and Topological Properties of All the Reconstructed PTLs^a^

PTL	ε	*r*_fiber_	β	*r*_m–pore_	*d*_max_	*R*_m_	*R*_q_	*R*_SA_ (10 μm)
unit	%	μm		μm	μm	μm	μm	m^2^/m^2^_geo_
P44	44.0	5.0	10,000	7.5	110.0	8.3	13.0	1.04
P54	54.0	5.0	10,000	10.4	141.0	7.7	14.2	0.99
P64	64.0	5.0	10,000	14.6	137.0	18.0	24.9	0.63
P74	74.0	5.0	10,000	24.9	200.0	18.6	27.7	0.57
P84	84.0	5.0	10,000	41.1	200.0	31.1	46.3	0.50
R3	74.0	3.0	10,000	12.3	169.0	10.8	18.2	1.45
R4	74.0	4.0	10,000	17.8	181.0	14.8	24.3	0.98
R5	74.0	5.0	10,000	24.9	200.0	18.6	27.7	0.57
R6	74.0	6.0	10,000	26.3	200.0	28.0	43.4	0.54
B2	74.0	4.0	2	13.4	200.0	24.8	36.3	0.73
B5	74.0	4.0	5	15.1	200.0	19.2	30.4	0.85
B10	74.0	4.0	10	15.5	200.0	22.1	32.7	0.76
B25	74.0	4.0	25	16.0	200.0	19.3	28.4	0.80
B100	74.0	4.0	100	16.8	192.0	13.1	22.6	1.02
B10000	74.0	4.0	10,000	17.8	181.0	14.8	24.3	0.98

a The porosity ε, anisotropy parameter β, pore *r*_m–pore_ and fiber radius *r*_fiber_, maximum surface depth *d*_max_, mean surface roughness *R*_m_, root-mean-square roughness *R*_q_, and specific surface area *R*_SA_ at a depth of 10 μm are characterized

### 3.2 Surface Roughness

An insufficient contact between different components, such as the interface of the PTL and CL, will introduce elevated interfacial resistances.^22^ Studies have demonstrated that optimizing the PTL/CL contact can effectively reduce the ohmic resistance,^12,48^ which reveals the importance of sufficient interfacial contact. However, there are very few studies on interfacial characterization and its correlation with PTL structures.

**Figure 3:**
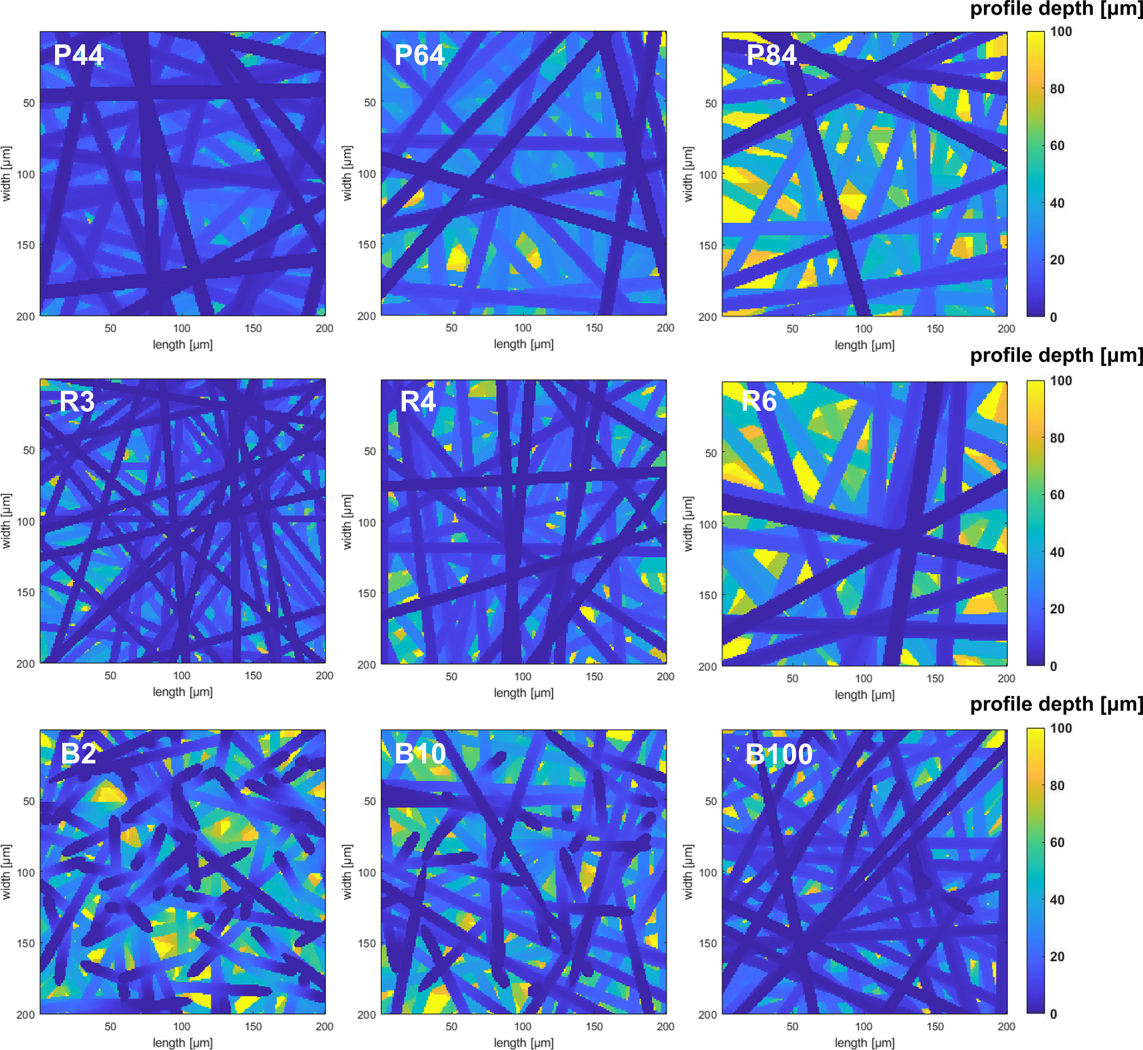
Depth profile maps for exemplary PTLs with different porosity (P44, P64, P84), fiber radii (R3, R4, R6), and anisotropy parameters (B2, B10, B100).

In order to select more desirable PTLs for minimizing the interfacial resistances in practical applications, it is necessary to elucidate the surface characteristics of PTLs with different structural features. Figure 3 shows the depth profile maps of exemplary PTLs P44, P64, P84, R3, R4, R6, B2, B10, and B100. The spatial distribution of fibers at different depths can be identified in all maps, and this distribution is influenced significantly by the PTL structure. Specifically, PTLs with lower porosity (such as P44) exhibit a denser distribution and thus a flatter PTL surface. As the porosity increases, the fibers distribute more sparsely, and the maximum surface depth *d*_max_ increases. Consequentially, P84 exhibits a sparser porous structure and a coarser PTL surface. The same phenomenon occurs for the PTLs with different fiber radii. Compared to R6, R3, and R4 show denser structural characteristics and more sufficient superficial contact. Additionally, R3 and R4 possess finely distributed fibers and therefore smaller local pore sizes. The effect of the anisotropy parameter is intrinsically different from the porosity and fiber radius. Small values of the anisotropy parameter (B2 and B10) imply a decreased angle between the fibers and the normal plane (i.e., the IP direction), which in turn exhibits uniformly distributed pores at the interface and a coarser surface. Previous studies have demonstrated that large open pores at the PTL/CL interface are a massive hindrance to mass transport.^36^ Under the same oxygen inlet conditions (11% surface coverage), the PTL with larger open pores at the interface exhibited a two-phase permeability of about 1.5 × 10^–13^ m^2^ and an oxygen breakthrough points of 1.1, while the PTL with smaller pores exhibited a two-phase permeability of about 5.2 × 10^–13^ m^2^ and an oxygen breakthrough points of 20, demonstrating a significant enhancement. Hence the uniformly distributed small pores on the interface are expected to facilitate the timely removal of oxygen generated on the CL and the supply of water from the flow channels in the electrolysis cell.

Furthermore, Table 2 lists the mean surface roughness *R*_m_ and the root-mean-square roughness *R*_q_ of all PTLs, which also show a strong correlation with PTL morphological properties. The increase in the porosity and fiber radius and the decrease in the anisotropy parameter lead to a greater roughness of the PTL, i.e., a coarser surface. The roughness of P84 is almost four times that of P44, and the roughness of R6 is as much as twice that of R3. In contrast, the effect of β on the roughness is less dramatic, and an opposite trend is detected with increasing β. For PTLs with different porosity and fiber radii, *r*_m–pore_ and *R*_m_/*R*_q_ are positively correlated, while for PTLs with different anisotropy parameters, they are negatively correlated. This relation is attributed to the smaller β values leading to a 3-D distribution of fibers, rather than a 2D distribution. By checking the pore size distribution of the PTLs with different anisotropy parameters in Figure S2 (Supporting Information), the spatially distributed fibers discrete the entire pore space into numerous small pores (B2), which in turn leads to a lower *r*_m–pore_. These findings provide broad insights for further weighing ohmic resistance and mass transport resistance. Combined with the adjustment of the overall bulk property of PTLs by the porosity and fiber radius, lowering the anisotropy leads to only a small loss of surface flatness, but is expected to facilitate the removal of oxygen and the supply of water at the PTL/CL interface.^36,59^

### 3.3 Specific Surface Area

**Figure 4:**
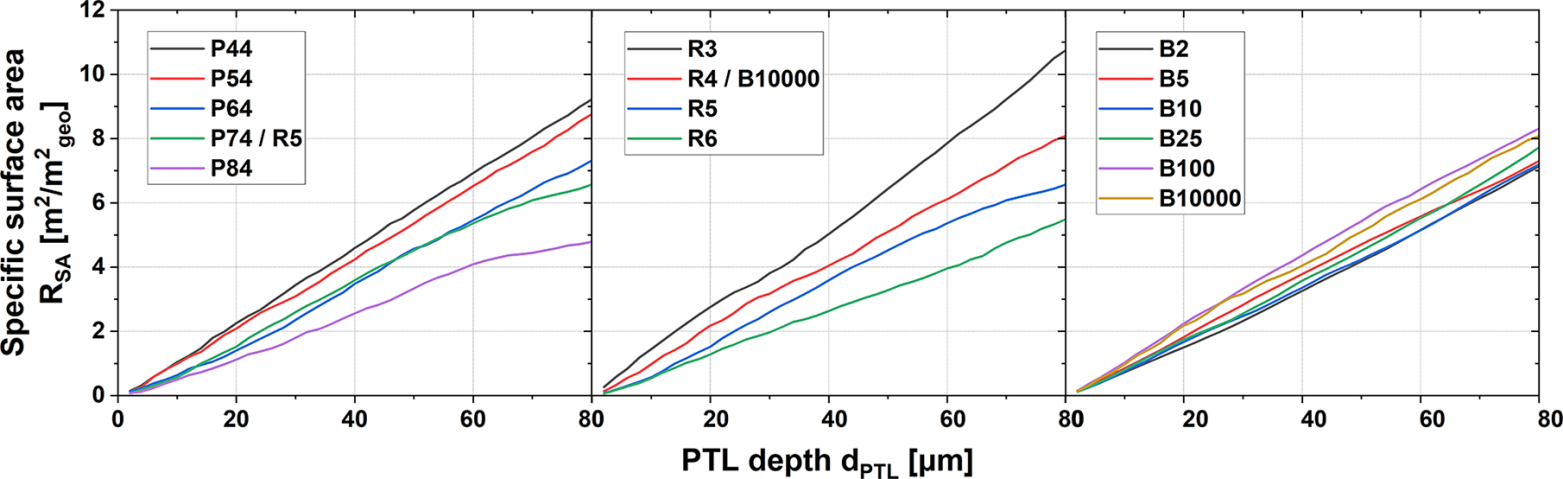
Specific surface area *R*_SA_ of all PTLs considered in this work as a function of the position along the PTL depth *d*_PTL_.

In practical applications, the cell components are held together under pressure, which in turn leads to the deformation of the membrane electrode assembly (MEA) and an intrusion of the CL into the PTL (see Figure 2b). The specific surface area *R*_SA_ helps to predict the variation in potential accessible interfacial area for different PTLs. Although it does not represent real interfacial contact, it is independent of the membrane and the CL, indicating the inherent characteristics of the PTL. For the same membrane and CL, a higher *R*_SA_ may lead to more potential contact. The values of *R*_SA_ as a function of the PTL depth *d*_PTL_ of all PTLs were determined and are shown in Figure 4. As the *d*_PTL_ within the observation range increases, the *R*_SA_ values of all PTLs increase almost linearly but at different growth rates. A trend can be seen that PTLs with lower porosity, lower fiber radii, and higher anisotropy parameters show higher *R*_SA_ values, i.e., superior interfacial contact. By a more detailed comparison, the fiber radius has the most significant effect on the interfacial contact, followed by the porosity, and finally the anisotropy parameter. As can be seen from Table 2, the *R*_SA_ (10 μm) of P44 is twice that of P84, the *R*_SA_ (10 μm) of R3 is 2.6 times that of R6, while the *R*_SA_ (10 μm) values of the B-series PTLs do not vary significantly. Therefore, it can be predicted that a PTL with a porosity of 0.44, a fiber radius of 3 μm, and an anisotropy parameter of 10,000 will exhibit the highest *R*_SA_, i.e., the optimal contact between the PTL and the CL.

From the above results, it is found that different structural features of PTLs have a significant impact on their topology. For a given fixed fiber radius, the PTLs with a lower porosity feature a denser structure and better interfacial contact. The same conclusion applies to PTLs with smaller fiber radii at the same porosity. The anisotropy parameter has a relatively moderate effect on the topological and morphological properties of PTLs, with larger values (anisotropic PTLs) corresponding to larger mean pore sizes and better interfacial contacts. Therefore, the porosity and fiber radius should be the primary parameter in the design or application of PTLs, and these two parameters should be selected according to different operating conditions and supplemented by the adjustment of anisotropy parameters for fine-tuning of the structures.

It should be noted that the selection of PTLs should be subject to real application conditions. In this work, only the basic properties of PTLs that have not been assembled in actual equipment are described. In practical cases, PTLs are operated under pressure, so PTLs with appropriate porosity/fiber characteristics, etc., should be selected to ensure adequate mechanical stability. It is also important to emphasize that PTLs must be matched to the membrane properties. For example, when thinner membranes are adopted, consideration should be given to the surface roughness and surface depth of the PTL in order to avoid serious deformation of the membrane, which could lead to its failure.^13^

### 3.4 Effective Gas Diffusion Coefficient and Tortuosity

The transport properties of PTLs are important for PEM electrolyzer operations, as they may contribute significantly to the mass transport and ohmic losses depending on their varied properties.^33,47^ The link between PTL structures and transport properties needs to be reported urgently in order to provide more effective guidelines for the design and application of PTLs.

To minimize statistical errors, three PTLs with the same structural parameters were reconstructed. The transport properties of each PTL were calculated by PSM and the average was reported. In the following, the transport properties of all PTLs including the gas diffusion coefficient, tortuosity, electrical conductivity, and thermal conductivity will be reported and discussed, as well as the potential links between the structural and transport properties.

**Figure 5:**
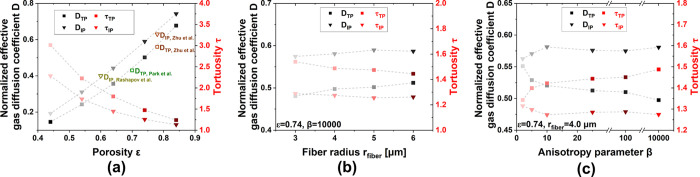
Predicted normalized effective gas diffusion coefficient and tortuosity in IP and TP directions of PTLs with different (a) porosity, (b) fiber radii, and (c) anisotropy parameters. Lines are added for the reader as guide for the eye only and are not meant to represent a trend with physical meaning.

The normalized effective gas (here oxygen) diffusion coefficient and tortuosity in the IP and TP directions of PTLs as functions of the porosity, fiber radii, and anisotropy parameters are shown in Figure 5. Figure 5a indicates that the gas diffusion coefficient of PTLs with different porosity calculated by PSM is in good agreement with the numerical calculations and experimental results from the literature.^50,60,69^ An increase in the porosity leads to an increase in the gas diffusion coefficient both in the IP and TP directions. The IP direction features a higher diffusion coefficient compared to the TP direction. An increase in the porosity from 0.44 to 0.84 results in a 2.8-fold increase in the IP gas diffusion coefficient and a 3.6-fold increase in the TP gas diffusion coefficient. The diffusivity in the IP direction is also important because it plays a key role in the in-plane transport within the PTL, especially in the location of the ribs under the flow field. Equation 9 demonstrates that the tortuosity τ is inversely related to the normalized effective gas diffusion coefficient *D*. A higher *D* indicates a lower τ, i.e., the more direct transport of gas or liquid.

While the porosity shows a significant impact on the gas diffusion coefficient and the tortuosity, the fiber radius and the anisotropy parameter feature a relatively minor effect, as shown in Figure 5b,c. The increase in fiber radius leads to an increase in the gas diffusion coefficient of only 6.5% in the TP direction, while almost no changes in the IP direction. Although only a slight effect is exhibited, the results still suggest that a large fiber radius facilitates the transport of gas and leads to a less porous structure. When the fibers are all stacked in-plane, the PTL features a strong anisotropy, and the transport properties in the IP direction are superior to those in the TP direction. In the PEM electrolyzer, the TP transport properties dominate the species transport within the PTL at almost all times. Therefore, it is expected that the TP transport properties can be enhanced by adjusting the fiber orientation. The results in Figure 5c confirm this prediction. A small anisotropy parameter (β = 2) enhances the diffusion in the TP direction and leads to a more homogeneous structure (almost equal tortuosity in IP and TP directions). PTLs with different anisotropy parameters featuring a porosity of 0.74 and a fiber radius of 4 μm, lead to an already relatively high diffusion coefficient of this series of PTLs. The adjustment of β from 10,000 to 2 brings a 10% further improvement in diffusion coefficient surprisingly. Moreover, it can be predicted that a further decrease of the anisotropy parameter (β tends to zero) will lead to a further enhancement of the diffusion in the TP direction.

### 3.5 Electrical and Thermal Conductivity

The gas diffusion is associated with the void space in the PTL, while the conductance of electrons and heat depends mainly on the solid material and fiber connections. Therefore, conductance may behave differently as gas diffusion if the PTL structure is changed. Evaluating the variance in electrical conductivity of different PTLs helps in developing structures with low ohmic resistances while optimizing the thermal conductivity facilitates better thermal management.

**Figure 6:**
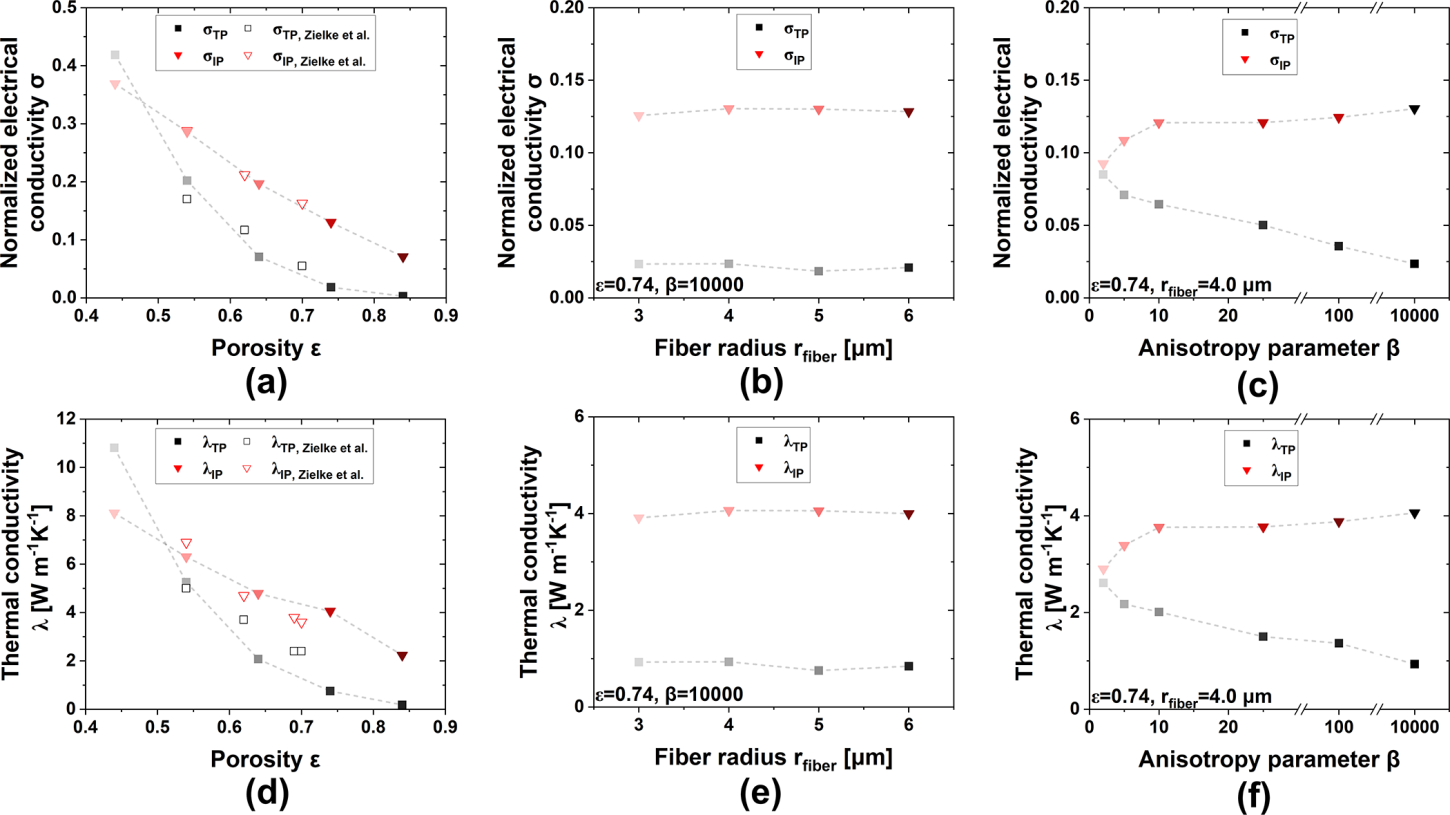
Electrical and thermal conductivity in IP and TP directions of PTLs with different (a,d) porosity, (b,e) fiber radii, and (c,f) anisotropy parameters. Lines are added for the reader as guide for the eye only and are not meant to represent a trend with physical meaning.

The electrical and thermal conductivity in the IP and TP directions of PTLs as functions of the porosity, fiber radii, and anisotropy parameters are shown in Figure 6. It should be noted that the electrical conductivity only reflects the effect of the internal microstructure, and the interfacial resistance is not included. We also used some experimental measurements of the electrical and thermal conductivity of PTLs with different porosity to verify the validity of the method, as shown in Figure 6a,d.^10^

Both the IP electrical conductivities and the IP thermal conductivities show almost perfect agreement with experimental measurements, with only a slight underestimation of the electrical and thermal conductivity in the TP direction. We attribute this slight deviation to the reduced number of contacts caused by the crossing and overlapping of fibers in the reconstruction process. In addition, it should be noted that since a fiber orientation almost exactly parallel to the IP direction (β = 10,000) was adopted in the study of the impact of porosity, the influence of the fiber orientation was almost completely isolated. In other words, the fibers of the real PTL inevitably are at an angle to the IP direction and thus more sufficiently connected in the TP direction, which in turn leads to relatively a little higher electrical and thermal conductivity. Therefore, the thermal conductivity at about β = 15 in Figure 6f is consistent with this deviating experimental result, which illustrates the potential contribution of fiber orientation to the electrical and thermal conductivity of the PTL. In conclusion, the PSM provides excellent reliability and these results can provide comprehensive insights into the impact of different structural parameters.

As shown in Figure 6a,d, the electrical and thermal conductivity decrease with increasing porosity, and both the electrical and thermal conductivity in the IP direction are higher than those in the TP direction at higher porosity (>0.5). We presume that the low-porosity PTL consists of more fibers, so there is more adequate contact between the horizontally oriented fibers in different layers, providing more adequate conduction in the TP direction. The electrical and thermal conductivity in the IP direction are significantly linearly related to the porosity.

Figure 6b,e indicates that the PTLs with smaller fiber radii feature higher electrical and thermal conductivity, even though the effect of fiber radius on the conductivities is very slight. Unlike the gas diffusion coefficient, the electrical and thermal conductivity are more strongly influenced by the anisotropy parameter. The anisotropy parameter is reduced from 10,000 to 2 (from an anisotropic to a more isotropic structure), while the electrical and thermal conductivity in the TP direction increase almost four times and three times respectively, and respectable conductivities still remain in the IP direction. This finding elucidates the importance of fiber orientation. Although studies have explained the impact of fiber orientation on IP transport properties,^69^ its potential contribution to TP transport properties is reported for the first time in this work to the best of our knowledge. The enhanced electrical and thermal conductivity will undoubtedly enhance the electrical and thermal conduction in the TP direction of the PTL.

From the above PSM results, it is found that different structural features of PTLs have a significant impact on transport properties. For a given fixed fiber radius, the PTL with a lower porosity features a higher tortuosity, electrical, and thermal conductivity. The same conclusion applies to PTLs with smaller fiber radii at the same porosity. The anisotropy parameter has a significant effect on the transport properties of PTLs, and lower values are expected to significantly promote mass transport and enhance electrical and thermal conductivity. Therefore, the porosity and anisotropy parameters should be the main criteria for PTL applications from the perspective of transport properties.

The results of the transport properties of all PTLs are listed in Table 3. For electrolyzer plants, the electrical and thermal conductivity are usually of more interest, in addition to the two-phase transport within the PTL. The present work aims at providing these parameters and the corresponding correlations for the reference of researchers and manufacturers. The corresponding results can be utilized to provide a first prediction of the transport performance of different PTLs so that the most suitable PTLs can be selected in a more coordinated manner to match different electrolyzer assemblies and operating conditions. This knowledge gained will help to optimize the development of electrolyzers in the end.

**Table 3:** Tortuosity τ, Normalized Effective Gas Diffusion Coefficient *D*, Normalized Electrical Conductivity σ, and Thermal Conductivity λ Computed for the PTLs in Both IP/TP Directions

PTL	unit	P44	P54	P64	P74	P84	R3	R4	R5	R6	B2	B5	B10	B25	B100	B10000
τ (IP/TP)		2.29/3.01	1.74/2.23	1.45/1.80	1.26/1.47	1.13/1.24	1.29/1.54	1.27/1.49	1.26/1.47	1.26/1.45	1.32/1.34	1.30/1.40	1.27/1.42	1.29/1.44	1.29/1.45	1.27/1.49
*D* (IP/TP)	%	19.24/14.60	31.09/24.26	44.22/35.59	58.96/50.18	74.24/67.73	57.44/48.07	58.07/49.75	58.96/50.18	58.67/51.20	56.27/55.10	57.05/52.89	58.13/52.05	57.58/51.26	57.49/51.01	58.07/49.75
σ (IP/TP)	%	36.91/41.89	28.64/20.22	19.68/7.07	13.01/1.84	7.09/0.30	12.56/2.33	13.04/2.35	13.01/1.84	12.83/2.09	9.25/8.51	10.86/7.10	12.07/6.45	12.08/5.02	12.44/3.57	13.04/2.35
λ (IP/TP)	W/mK	8.12/10.81	6.31/5.26	4.79/2.08	4.06/0.75	2.24/0.19	3.91/0.93	4.06/0.93	4.06/0.75	4.00/0.84	2.90/2.61	3.39/2.18	3.76/2.01	3.77/1.50	3.88/1.36	4.06/0.93

Taking into account the PTL topological, morphological, and transport properties, the application of PTL structures should be differentiated according to the operating conditions. When the electrolyzer is operated at low current densities (nonstarvation conditions), the PTLs should feature a low porosity and small fiber radius to enhance the interfacial contact and electrical and thermal conductivity, and a low anisotropy parameter to further improve electrical and thermal conductivity. When it is operated at high current densities (starvation conditions), the PTLs should feature a high porosity to enhance mass transport, a low anisotropy parameter to improve electrical and thermal conductivity, and a small fiber radius to improve interfacial contact. Further, the graded PTLs with different structural features per layer are strongly recommended to adequately balance the topological, morphological, and transport properties. The combined integration of the required different features into a graded PTL promises a comprehensive trade-off between interfacial resistances, ohmic losses, and mass transport losses.

The above results expose the great potential of the anisotropy parameter, i.e., the fiber orientation, for improving the transport properties of PTLs, especially in the TP direction. In most cases, high values for gas diffusion coefficient and electrical and thermal conductivity in the TP direction are preferred. Hence, the effect of structural features on the heterogeneity of PTLs is necessary to be elaborated.

**Figure 7:**
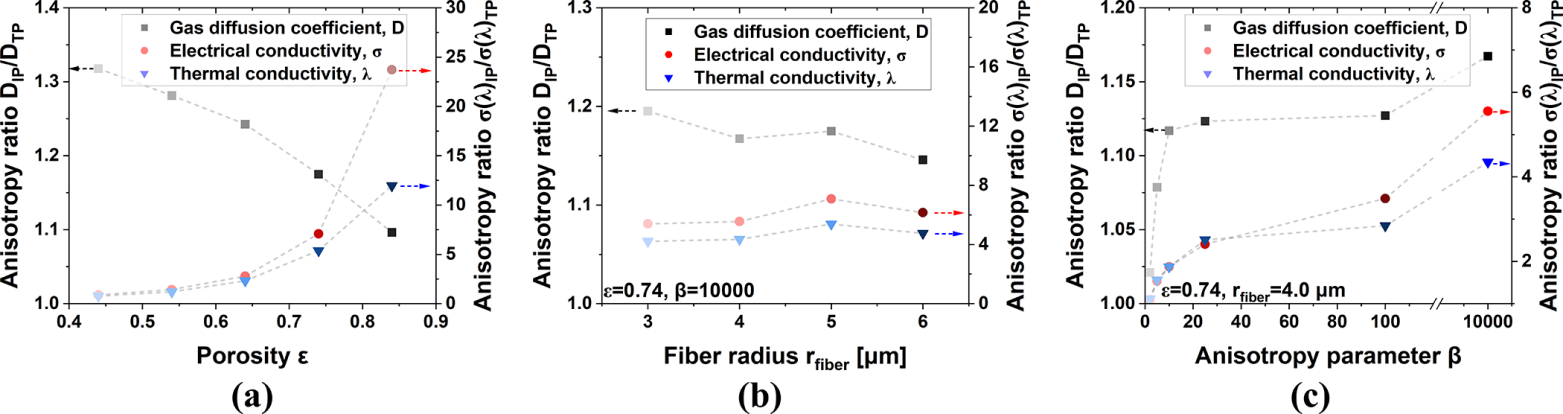
Anisotropy ratio of the transport properties of PTLs with different (a) porosity, (b) fiber radii, and (c) anisotropy parameters. Lines are added for the reader as guide for the eye only and are not meant to represent a trend with physical meaning.

The anisotropy ratio of the transport properties of PTLs as functions of the porosity, fiber radii, and anisotropy parameters is shown in Figure 7. The results show that changing the fiber radius (Figure 7b) does not cause a very significant effect on the anisotropy ratio, while an increase in the porosity and anisotropy parameter (Figure 7a,c) leads to a severe anisotropy. Specifically, as the higher porosity of fiber-based PTLs leads to already higher IP and TP gas diffusion coefficients, changing the structural properties, especially the fiber radius, does not drastically change the anisotropy ratio of the gas diffusion coefficient, as shown in Figure 7b,c.

However, the anisotropy ratio of the electrical and thermal conductivity is more strongly influenced by the PTL structure. As shown in Figure 7a, the anisotropy ratio of the electrical and thermal conductivity is relatively small at a low porosity, which is attributed to more adequate contact in the TP direction. However, as the porosity increases, the contact decreases and the anisotropy ratio increases rapidly. The increased anisotropy ratio means that the electrical and thermal conductivity change at a faster rate in the TP direction compared to the IP direction, which is catastrophic for the electrolyzer operation due to the inefficient electrical conduction in the TP direction. It is promising that lowering the β value can decrease the anisotropy ratio and compensate for the losses of electrical and thermal conductivity in the TP direction, as shown in Figure 7c.

### 3.6 Graded PTLs Targeted at Enhanced Properties

**Figure 8:**
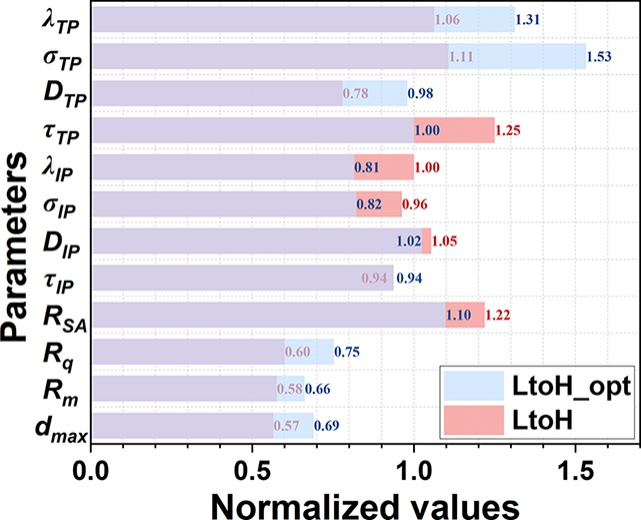
Normalized topological and transport properties of the graded (LtoH and LtoH_opt) PTLs. The maximum surface depth *d*_max_, mean surface roughness *R*_m_, root-mean-square surface roughness *R*_q_, and specific surface area *R*_SA_ at a depth of 10 μm are listed. The tortuosity τ, effective gas diffusion coefficient *D*, effective electrical conductivity σ, and thermal conductivity λ in both IP/TP directions are shown.

In order to improve the topological and transport properties of the PTL, thus reducing the structure-related mass transport losses and ohmic losses, we proposed graded PTLs with customized structural properties. The PTL configuration for the low porosity region adjacent to the CL and the high porosity region close to the flow channels is denoted as “Low to High” (“LtoH”). Another optimized PTL structure with the same porosity gradient as “LtoH” (0.44–0.74 from the CL to flow channels) but with a different fiber radius and orientation was reconstructed and noted as “Low to High, optimized” (“LtoH_opt”), as shown in the subplot (Graded PTLs) of Figure 1. These graded PTLs were obtained by merging two layers of PTLs of the same thickness with different porosity into one layer and finally featured an overall porosity of 0.59. In addition, a single-layer PTL with a porosity of 0.59 is employed for comparison and is denoted as “SL”. The electrical and thermal conductivity of the SL are predicted by the fitted curves in Figure S3 (Supporting Information), and the other parameters of the SL are linearly predicted. To show the differences clearly, Figure 8 presents the normalized results after dividing the parameters of the graded PTLs by the parameters of the SL (all the parameters of the SL are 1).

The results indicate that the graded PTLs (LtoH and LtoH_opt) show enhanced structural and transport properties over the single-layer PTL (SL). Both LtoH and LtoH_opt feature a smaller surface roughness and a higher contact area. The transport properties of LtoH and SL are comparable, while the transport properties of LtoH_opt, especially the electrical and thermal conductivity in the TP direction, are greatly improved. Moreover, in our previous study, we demonstrated that LtoH_opt also exhibits optimal oxygen transport capacity.^59^ This finding confirms the great potential of the graded PTLs in practical electrolyzers. Furthermore, the comparison of the graded PTLs LtoH and LtoH_opt reveals the contribution of fiber orientation. Despite the application of a larger fiber radius in LtoH_opt, it still shows significantly improved TP transport properties compared to LtoH. In combination with the aforementioned studies, it can be predicted that further lowering the fiber radius of LtoH_opt will result in currently optimal structural and transport properties. Similarly, these relationships or configurations can be generalized for research on XCT characterization of relevant porous media in other batteries to optimize the research cost and improve performance, such as the prediction of the interfacial area and the optimization of the porous structure.^70,71^

## 4 Conclusions

We employed stochastic reconstruction and pore-scale modeling to investigate the effects of the morphology on the topological and transport properties of PTLs in PEM electrolyzers. Specifically, various PTLs with different porosity, fiber radii, and anisotropy parameters were reconstructed. Subsequently, the morphological properties, such as the mean pore radius, and the topological properties including the surface roughness and specific surface area, etc. were numerically characterized and the underlying relationship between the morphology and topology was revealed. Then, the pore-scale modeling method was applied to calculate the transport properties of all PTLs, including the effective gas diffusion coefficient, tortuosity, and electrical and thermal conductivity. We described the link between the transport properties and the morphological properties of the PTLs and presented a relation between the electrical and thermal conductivity in the through-plane direction as a function of the porosity. Based on the gained understanding and the identified relation, we reconstructed two PTLs with customized structures, e.g., having gradients of porosity, to indicate, for example, experimentalists and manufacturers what an optimal PTL could look like.

The results indicate that the transport properties of PTLs are sensitive to variations in porosity and anisotropy parameters, while the topological properties are mainly dominated by fiber size and porosity. PTLs with lower porosity, smaller fiber radii, and higher anisotropy parameters feature a lower surface roughness and higher specific surface area, i.e., generally more adequate interfacial contact and lower interfacial resistances. Similarly, PTLs with lower porosity and smaller fiber radii generally have higher electrical and thermal conductivity, while lowering the anisotropy parameter will further enhance the electrical and thermal conductivity substantially in the through-plane direction. This finding implies that a significant change to the transport properties can be evoked by customizing the fiber orientation. The electrical and thermal conductivity in the through-plane direction are approximated as a logistic function of the porosity, with the fitted curves featuring an inverse S-shape. Graded PTLs with enhanced structural and transport properties can be developed while the porosity, fiber radius, and fiber orientation are fully customized. Herein, we provide a substantial framework for exploring the transport properties of PTLs and comprehensive insights into the optimization of the design and manufacture of PTLs. Customized graded PTLs create more possibilities for accelerating the commercialization of PEM electrolyzers and improving the performance of the next generation of PEM electrolyzers.

## Supporting Information Available

Schematic illustration of the anisotropy parameter, PSD of the PTLs, and fitted curves for the electrical and thermal conductivity of the PTLs as a function of the porosity (PDF)
