# Supplementary material for: Predicting the Topological and Transport Properties in Porous Transport Layers for Water Electrolyzers
Source: ACS Appl Mater Interfaces. 2023 Nov 10;15(46):54129–42. doi: 10.1021/acsami.3c12345 (PMC10688607; doi:10.1021/acsami.3c12345)
Supplement: Supplementary file 1 — am3c12345_si_001.pdf [file am3c12345_si_001.pdf]

Supporting Information

Predicting the topological and transport properties in porous transport layers for water electrolyzers

Jiang Liu[a], *Felix Kerner*[a], *Nicolas Schlüter*[a], *and Daniel Schröder*[a,b*]

[a] Institute of Energy and Process Systems Engineering, Technische Universität Braunschweig, Langer Kamp 19B, 38106 Braunschweig, Germany

[b] Battery LabFactory Braunschweig (BLB), Technische Universität Braunschweig, Langer Kamp 19, 38106 Braunschweig, Germany

*Corresponding authors: d.schroeder@tu-braunschweig.de

The anisotropy parameter β (a positive value used to customize the orientation of fibers) was introduced in the stochastic reconstruction algorithm. As shown in Figure S1, when $\beta \rightarrow 1$, the most fibers will be evenly distributed throughout the whole domain, with most fibers at a 45° angle to the IP; when $\beta \rightarrow +\infty$, most fibers will be stacked in the IP; and when $\beta \rightarrow 0$, more fibers will be stacked in the TP, perpendicular to the CL.

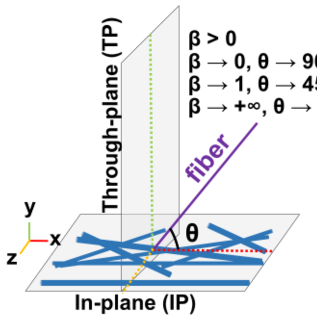

Figure S1. Schematic illustration of the fiber orientation and their relative angle θ, which governs the anisotropy parameter β.

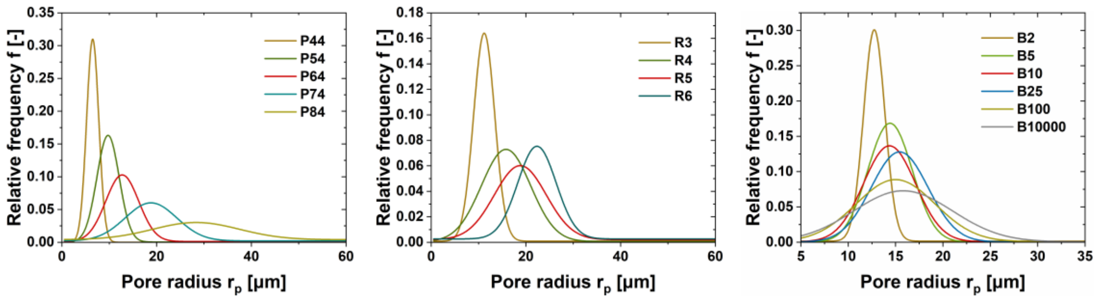

Figure S2. The pore size distribution of the PTLs with different porosity (P44, P54, P64, P74, P84), fiber radii (R3, R4, R5, R6), and anisotropy parameters (B2, B5, B10, B25, B100, B10000), adapted from Liu et al.[59]

To provide more insights into the electrical and thermal conductivities of PTLs, the fitted curves for electrical and thermal conductivities in the TP direction of PTLs as a function of the porosity are shown in Figure S3, with blue color indicating the electrical conductivity and red color indicating the thermal conductivity. The two fitted curves feature an inverse S-shape. The changes of electrical and thermal conductivity in the TP direction with porosity can be divided into three stages. Firstly, in the porosity range of 0-0.3, the electrical and thermal conductivity remains at a very high value and

decreases slowly; then, in the porosity range of 0.3-0.6, the increase in the porosity leads to a significant decrease in the electrical and thermal conductivity; and finally, in the porosity range of 0.6-1, the electrical and thermal conductivity is already at a very low level and continues to decrease slowly. The result illustrates the dramatic effect of porosity on the electrical and thermal conductivity of PTLs.

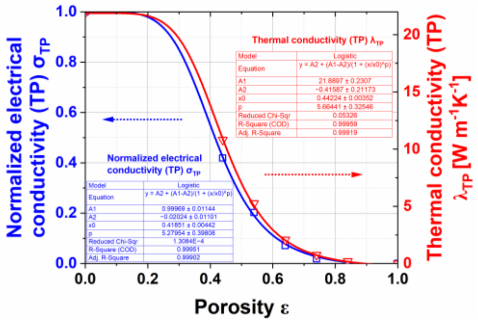

Figure S3. The fitted curves for the electrical and thermal conductivities of the PTLs as a function of the porosity.
